# Exploring the causal impact of mitochondrial dysfunction on epilepsy: a mendelian randomization study

**DOI:** 10.1590/1414-431X2025e14524

**Published:** 2026-03-09

**Authors:** Lin-Ming Zhang, Fei Wang, Bing-ran Zhang, Qiu-juan Zhang, Yan-lin Zhu, Shu-ji Gao, Hao Wang, Ming-wei Liu

**Affiliations:** 1 Department of Neurology, The First Affiliated Hospital of Kunming Medical University, Kunming, Yunnan, China; 2 Department of Neurosurgery, The First Affiliated Hospital of Kunming Medical University, Kunming, Yunnan, China; 3 Department of Emergency, The First Affiliated Hospital of Kunming Medical University, Kunming, Yunnan, China; 4 Department of Nuclear Medicine, Sichuan Provincial People's Hospital, University of Electronic Science and Technology of China, Chengdu, Sichuan, China; 5 Department of Emergency, Dali Bai Autonomous Prefecture People's Hospital, Dali, Yunnan, China

**Keywords:** Epilepsy, Mitochondrial dysfunction, Mendelian randomization, OSBPL1A, HAGH, PANK2

## Abstract

Mitochondrial dysfunction contributes critically to epileptogenesis. Therefore, identifying key mitochondrial function-associated genes in epilepsy may provide novel insights into its pathogenesis. We employed expression quantitative trait loci (eQTLs) and Mendelian randomization analyses to assess mitochondrial-epilepsy causality, with leave-one-out validation confirming the reliability and directionality of the results. The results revealed that hydroxyacylglutathione hydrolase (*HAGH*), oxysterol-binding protein-related protein 1A (*OSBPL1A*) and pantothenate kinase 2 (*PANK2*) were pivotal epileptogenic genes. *HAGH* modulates the mechanistic target of rapamycin complex 1 (mTORC1) signaling and fatty acid metabolism pathways. *OSBPL1A* mediates apoptotic and reactive oxygen species (ROS) pathways. *PANK2* regulates phosphatidylinositol 3-kinase (PI3K)/protein kinase B (AKT)/mammalian target of rapamycin (mTOR) signaling and Notch signaling cascades. Additionally, these genes participate in inflammatory pathways, including T cell receptor (TCR), mitogen-activated protein kinase (MAPK), and tumor necrosis factor (TNF) signaling. We demonstrated that *HAGH*, *OSBPL1A*, and *PANK2* constitute core pathogenic mechanisms in epilepsy. These genes potentially govern epileptogenesis through mitochondrial regulation via neuroinflammatory, immunomodulatory, and apoptotic pathways. Our findings provide a foundation for investigating epileptogenesis, discovering therapeutic targets, and identifying prognostic biomarkers.

## Introduction

Epilepsy is a prevalent neurological disorder affecting individuals across all age groups and geographic regions globally. The World Health Organization estimates that approximately 50 million people live with epilepsy worldwide, with over 4 million new cases annually and an incidence rate of 61.4 per 100,000 people (1,2). Over 80% reside in developing countries, with annual increases of 4.5×10^5^ cases per year (3). The prevalence of active epilepsy ranges from 2.70-7.10% in high-income countries, 3.70-22.20% in middle-income countries, and 2.20-16.00% in low-income countries, indicating normal distribution patterns (3,4). In China, the incidence of epilepsy is 5-7%, corresponding to 6.5-9.1 million affected individuals. The bimodal age distribution shows a peak incidence in adolescent and elderly populations (5).

This chronic disorder features transient brain dysfunction due to sudden neuronal hyperexcitability, which confers enduring seizure susceptibility. Epilepsy is characterized by recurrent seizures and has heterogeneous etiologies and clinical presentations. As one of the most prevalent neurological disorders, it affects all demographics, regardless of age, race, socioeconomic status, or geography (6). Cumulative evidence has demonstrated that seizure progression is correlated with recurrent neuronal injury from persistent epileptiform activity. Ictal events induce neuronal damage, with hippocampal neurodegeneration playing pivotal roles in epileptogenesis. Consequently, postictal neuroprotection is crucial for preventing seizure recurrence and progression. Thus, optimal management requires both seizure control and neuroprotective strategies to mitigate postictal injury. The prognosis varies by etiology, onset age, and seizure type; approximately 70% of patients achieve medication-controlled remission (7). Comprehensive management encompasses pharmacotherapy, lifestyle modification, and surgical interventions. This disorder imposes substantial psychosocial burdens, necessitating integrated therapeutic approaches. Therefore, elucidating epileptogenic mechanisms facilitates targeted therapy development and improved pharmacological control.

Mitochondria execute multiple critical functions, including cellular adenosine triphosphate (ATP) production, reactive oxygen species (ROS) generation, apoptosis regulation, and fatty acid metabolism (8). These interconnected mitochondrial functions are fundamental to maintaining normal brain activity (8). Mitochondria possess intrinsic quality control mechanisms to sustain overall health, including autophagy, biomechanical regulation, and biosynthetic processes. Impairment of these mitochondrial quality control processes results in mitochondrial dysfunction (8). For example, mitochondrial pathologies can induce intracellular calcium dysregulation, enhanced oxidative stress, and neuronal apoptosis, all of which facilitate epileptogenesis and seizure chronification (8). Direct evidence of mitochondrial dysfunction has been documented in certain forms of hereditary epilepsy (9). Epilepsy represents a prevalent comorbidity, affecting 40-60% of patients with mitochondrial disease and substantially compromising their quality of life (9). These findings suggest that mitochondrial function-targeted interventions could serve as effective therapeutic strategies for specific epileptic subtypes, particularly when conventional pharmacotherapy is inadequate. Therefore, elucidating the role of mitochondria at the onset and progression of epilepsy is critical for developing novel therapeutic strategies.

In recent decades, Mendelian randomization (MR) has been established as a robust methodology in epidemiological research. By capitalizing on Mendelian inheritance principles, MR employs genetic variants as instrumental variables to infer causal associations between exposures and outcomes (10). Consequently, MR has been applied to investigate the bidirectional associations between epilepsy and neuropsychiatric/neurological disorders (e.g., Alzheimer's disease, addictive behaviors, and disease-specific developmental-prognostic trajectories) (10,11). This framework mitigates false-negative/false-positive biases arising from unavoidable confounding and reverse causality inherent in traditional observational studies.

Driven by growing research interest in the association between mitochondrial dysfunction and epilepsy, the FinnGene database - a large genetic consortium focusing on mitochondrial disorders and epilepsy - has unveiled comprehensive genetic variant profiles (12). This study integrated weighted gene coexpression network analysis (WGCNA) data on mitochondrial dysfunction and epilepsy and employed MR methodology to address confounding biases and reverse causality. Potential genetic-level causal associations between mitochondrial dysfunction and epilepsy were evaluated to gain mechanistic insights into epileptogenesis and inform novel therapeutic strategies for epilepsy.

## Material and Methods

### Study design

The study design is shown in Supplementary Figure S1. First, we identified intersectional genes via WGCNA as candidate markers for Mendelian randomization analysis during the study follow-up. Second, key genes underlying causal associations with eQTL-derived outcomes were prioritized through MR analysis. Third, functional enrichment analysis of these key genes was performed via the Kyoto Encyclopedia of Genes and Genomes (KEGG) and Gene Ontology (GO) annotation frameworks. Pathway-specific regulatory mechanisms of these key genes were investigated via gene set variation analysis (GSVA) and gene set enrichment analysis (GSEA). Additionally, correlations between key gene expression levels and disease-associated genes were evaluated via the GeneCards database (https://www.genecards.org/).

### Sample exclusion criteria

Following the quality control protocols of the original study (13), RNA sequencing samples were filtered to retain only drug-naive epilepsy cases (n=34) and matched controls (n=51) in GSE143272. No additional exclusion of eQTLGen blood samples was performed.

### Data download

#### Exposure data

eQTL data were derived from the eQTLGen Consortium database (https://www.eqtlgen.org), which aims to characterize the genetic architecture of blood gene expression and decipher the genetic basis of complex traits. The large-scale eQTLGen project, which is currently in its second phase, focuses on performing genome-wide meta-analyses of blood samples.

#### Outcome data

The FinnGene database (https://www.finngen.fi/en) is a population-scale genetic research resource specializing in European populations that focuses on genetic diseases and population-specific genetic variants. The database contains geographically diverse genomic and phenotypic data, emphasizing gene-disease associations. It is particularly valuable for deciphering genetic applications in public health, notably in disease prevention and personalized medicine. FinnGene data serve as a critical resource for geneticists and epidemiologists, enabling deeper insights into the impact of genetic variants on health and disease. This dataset comprises 12,891 epilepsy cases (finngen_R10_G6_EPLEPSY) and 312,803 control individuals.

RNA-seq data were obtained from the National Center for Biotechnology Information (NCBI) Gene Expression Omnibus (GEO) database (https://www.ncbi.nlm.nih.gov/geo/), a repository specialized in archiving and distributing gene expression datasets. The epilepsy-associated dataset (GSE143272 Series Matrix File) was retrieved from the public GEO database, accompanied by the GPL10558 annotation file. This expression profile dataset included data from 85 individuals, with 51 in the control group and 34 in the disease group.

### WGCNA

By constructing a weighted gene coexpression network, coexpressed gene modules were identified to explore associations between gene networks and traits, as well as to pinpoint hub genes within the network. The WGCNA-R package (https://cran.r-project.org/package/WGCNA) was employed to construct a coexpression network for all genes, with the top 10,000 genes ranked by variance prioritized for downstream analysis. The weighted adjacency matrix was transformed into a topological overlap matrix (TOM) to evaluate network connectivity, followed by hierarchical clustering to generate the TOM matrix-based cluster tree. The divergent branches of the cluster tree represent distinct gene modules, with unique colors denoting separate modules. Genes were classified into modules via weighted correlation coefficients on the basis of expression profiles, where genes with similar expression patterns were grouped into the same module. This approach systematically partitioned all genes into distinct modules according to their expression dynamics.

### Mendelian randomized analysis

The FinnGene database (https://www.finngen.fi/en) integrates summary statistics from multiple GWASs (14). Epilepsy-associated eQTLs were extracted from FinnGene-derived outcome IDs. Single-nucleotide polymorphisms (SNPs) meeting genome-wide significance (P<1e-8) *per locus* served as potential instrumental variables (IVs). We evaluated the linkage disequilibrium (LD) between SNPs. Among SNPs with R2 values less than 0.001 (within a clumping window of 10,000 kb), only those with P values less than 5e-5 were retained for further analysis. The inverse-variance weighted (IVW) meta-analysis combined Wald estimates across SNPs to evaluate cumulative effects. Additionally, MR-Egger analysis (assuming InSIDE: instrument strength independent of direct effects), weighted median (robust to up to 50% invalid IVs), and weighted mode (higher power with reduced bias and type I error rate than MR-Egger) were applied. These four statistical methods enable comprehensive causal inference, especially when relying on a single analytical approach per SNP. This analysis aimed to estimate the effects of cis-acting variants and trans-regional gene expression in the whole blood of epilepsy patients. Causal link validation involved heterogeneity assessment (Cochran's Q test for IVW) and gene diversity analyses.

### GO and KEGG functional analyses

We performed functional annotation of the key genes via clusterProfiler to systematically characterize their biological significance (15). Functional categorization was conducted via the GO and KEGG frameworks (16). Enrichment analyses with statistical significance (P<0.05, q<0.05) in both the GO and KEGG pathways were considered significant.

### GSVA

GSVA constitutes a nonparametric, unsupervised methodology for evaluating gene set enrichment across transcriptomic profiles (17). This algorithm computes gene set enrichment scores by transforming gene-level differential expression into pathway-centric alterations, enabling systematic quantification of biological functions. Gene sets were obtained from the Molecular Signatures Database (version 7.0). We implemented the GSVA algorithm to systematically quantify pathway-level perturbations among sample cohorts, detecting potential biological functional alterations.

### GSEA

As previously described, individuals were stratified into high- and low-expression groups on the basis of gene expression levels. Signaling pathway variations between groups were then investigated via GSEA (18). Background gene sets (version 7.0) and annotated gene sets were derived from the Molecular Signatures Database (MSigDB), which serve as pathway-specific annotation references. Pathway-level differential expression analysis was performed, with significantly enriched gene sets (adjusted P<0.05) ranked by consistency scores. GSEA is typically used to explore the biological relevance of disease categorizations.

### Analysis of immune cell infiltration

CIBERSORT is a widely adopted computational method for deconvolving immune cell compositions in tissue microenvironments (19). Built on support vector regression (SVR) principles, it performs deconvolution of immune cell subtype expression matrices to quantify cellular proportions. The algorithm utilizes a panel of 547 biomarkers to distinguish 22 human immune cell phenotypes, including T cells, B cells, plasma cells, and myeloid subpopulations. In this study, the CIBERSORT algorithm was applied to analyze patient datasets, infer the relative proportions of 22 infiltrating immune cell types, and conduct Pearson correlation analysis between gene expression levels and immune cell abundances.

### Colocalization analysis

We employed the COLOC method and eQTL data (https://www.eqtlgen.org) to perform colocalization analysis with epilepsy GWAS datasets (https://opengwas.io/datasets). A 100-kilobase region flanking the index SNP was used to compute posterior probabilities. In the COLOC results, H3 denotes the posterior probability of two traits (gene expression and epilepsy) being correlated with distinct causal variants, whereas H4 represents the posterior probability of shared causal variants driving their association. A colocalization threshold of SNP.PP. H4>0.75 was applied.

### Analysis of the regulatory network of the key genes

This study utilized the RcisTarget R package (https://git.bioconductor.org/packages/RcisTarget) to predict transcription factors (TFs) and their regulatory networks. All computations in RcisTarget are fundamentally based on TF binding motifs (TFBMs). The normalized enrichment score (NES) of a motif is contingent on the total number of motifs in the reference database. In addition to the source data-annotated motifs, this study inferred additional annotation files through motif similarity analysis and gene sequence modeling. The initial step in assessing motif overrepresentation in gene sets is to compute the area under the curve (AUC) for each motif-gene set pair. This is computed via the motif sorting recovery curve derived from the gene set. The NES for each motif is computed from the AUC distribution of all the motifs across the gene set.

### Construction of the nomogram model

A nomogram, rooted in regression analysis, visually represents variable interrelationships within a predictive model by plotting scaled line segments proportional to gene expression levels on a unified plane. By constructing a multivariable regression model and assigning scores to each level of predictor variables according to their regression coefficients (reflecting contributions to the outcome) and summing these scores yields the total score, from which the predicted value is derived.

### Statistical analysis

MR analysis hinges on three core assumptions: 1) Relevance assumption: IVs are strongly associated with the exposure but have no direct effect on the outcome; 2) Independence assumption: IVs are independent of all confounding variables; 3) Exclusion restriction assumption: IVs influence the outcome exclusively through the exposure; gene pleiotropy is inferred if an IV affects the outcome via alternative pathways. Analyses were performed via R software (version 4.0.3, http://www.-project.org). All the statistical tests were two-sided, with statistical significance defined as P<0.05.

## Results

### WGCNA

**Figure 1:**
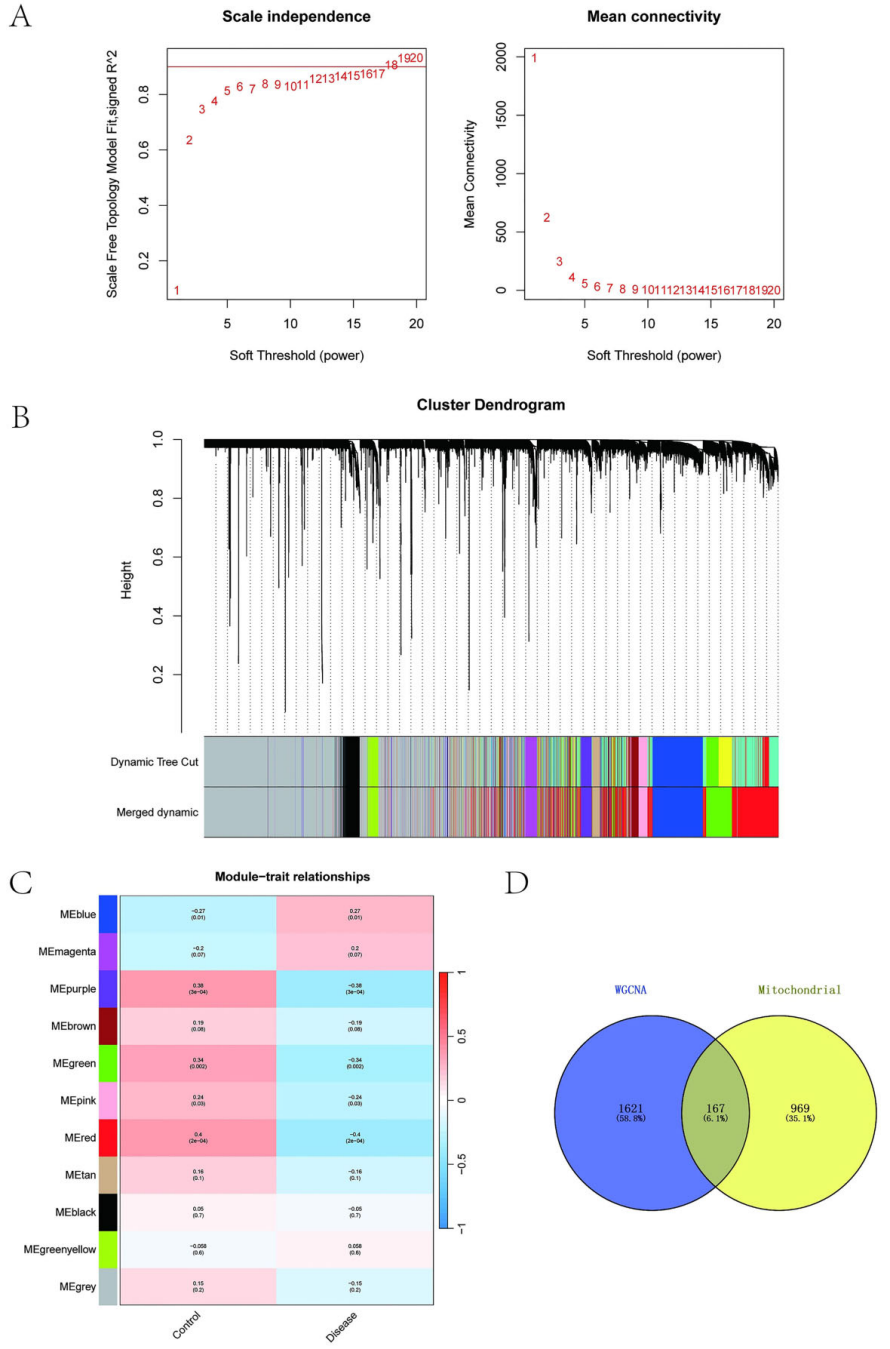
Weighted gene coexpression network analysis (WGCNA). **A**, Scale-free fit indices and mean connectivity across soft-thresholding powers. **B**, Hierarchical clustering dendrogram with color-coded modules. **C**, Module-trait correlation heatmap: blue (negative), red (positive). **D**, Venn diagram identifying key genes via the intersection of WGCNA module genes and mitochondrial genes.

The epilepsy-associated dataset (GSE143272) was downloaded from the GEO database. This dataset contains expression profiling data from 85 individuals, including 51 controls and 34 epilepsy patients. Using these expression data, a WGCNA was performed to identify disease-associated coexpression modules. A soft threshold of 12 was determined (refer to Figure 1A). Eleven gene modules were identified via TOM analysis, including black (343 genes), blue (1164 genes), brown (414 genes), green (776 genes), green yellow (202 genes), gray (4372 genes), magenta (246 genes), pink (299 genes), purple (236 genes), red (1788 genes), and tan (160 genes) modules. Module-trait correlation analysis revealed that the MEred module was most strongly correlated with epilepsy (cor=-0.4, P=2e-04), as shown in Figure 1B and C. Subsequently, 1,136 mitochondrial genes from the Human MitoCarta 3.0 database (https://www.broadinstitute.org/) were intersected with the 1788 genes in the MEred module. A total of 167 intersectional genes were identified (Figure 1D) and designated feature genes for subsequent MR analyses.

### Mendelian randomized analysis

**Figure 2:**
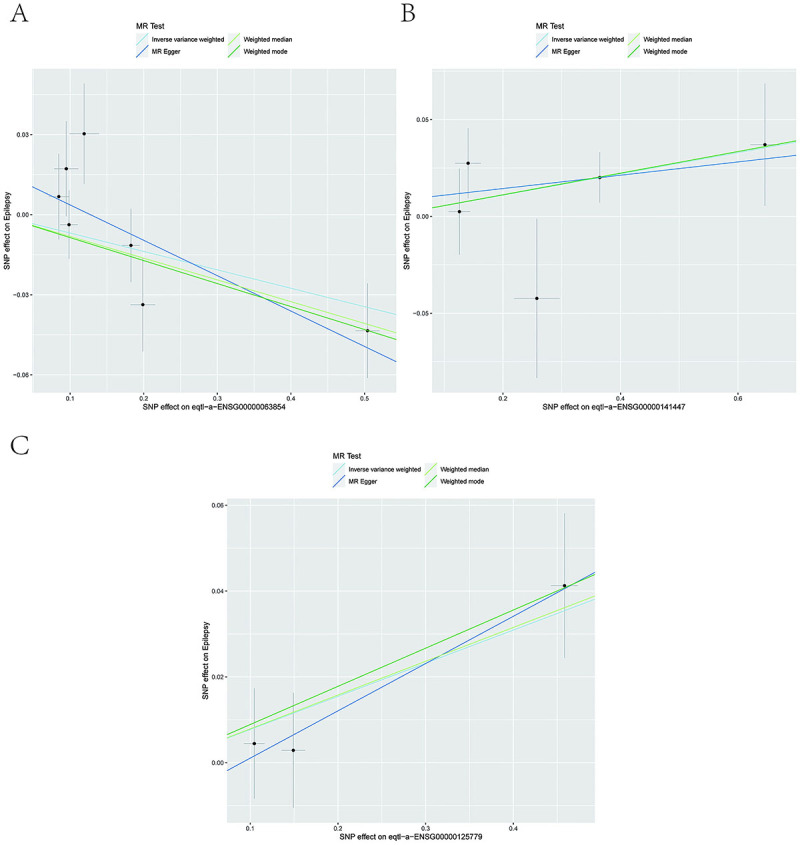
Mendelian randomization (MR) analysis. **A**-**C**, Scatter plots depicting MR associations between key genes and epilepsy. Regression line slopes indicate causal effects; colors represent statistical methods.

To pinpoint critical genes associated with epilepsy, we utilized a large-scale dataset comprising 325,694 individuals, including 12,891 epilepsy patients and 312,803 controls. Summary statistics revealed that the outcome dataset was Finngen_R10_G6_EPLEPSY. Using MR analysis, we selected instrumental variables and outcome data to derive causal associations for three eQTL-positive gene-outcome pairs (Figure 2; IVW, P<0.05). The identified genes were *HAGH*, *OSBPL1A*, and *PANK2*. *HAGH* was associated with a reduced epilepsy risk (0.933, 95%CI: 0.875-0.995, P=0.035), whereas *OSBPL1A* (1.056, 95%CI: 1.001-1.114, P=0.044) and *PANK2* (1.080, 95%CI: 1.013-1.152, P=0.018) were linked to increased risk. The robustness of these causal associations was evaluated via heterogeneity and sensitivity analyses. Heterogeneity analysis revealed no significant heterogeneity across the three gene-outcome associations (Supplementary Figure S2A-C; Q_ P>0.05). Leave-one-out sensitivity analysis revealed that removing any single SNP did not substantially change the effect estimates, indicating robust causal stability (Supplementary Figure S2D-F and Supplementary Figure S3A-C).

### Functional enrichment analysis of the key genes

**Figure 3:**
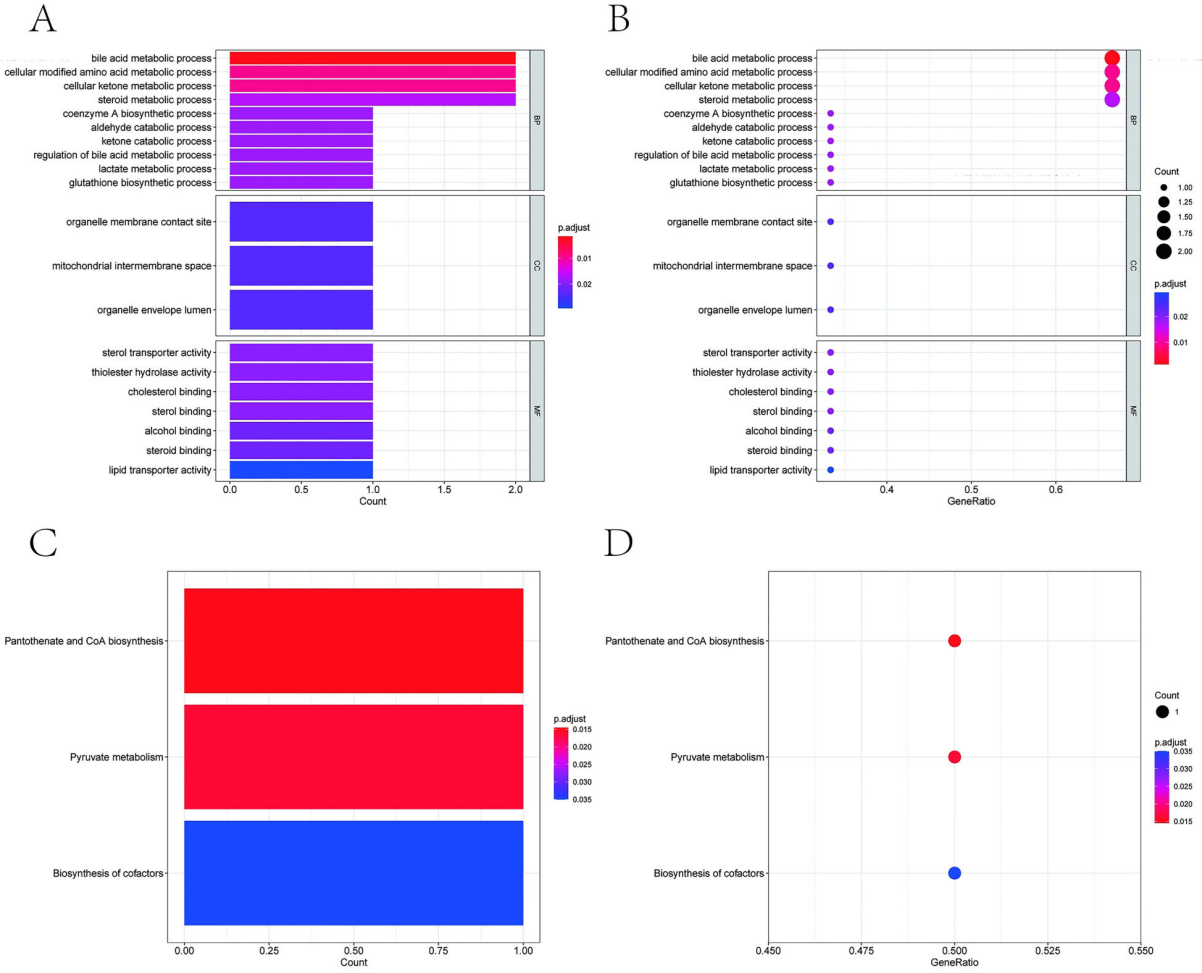
Enrichment analysis. **A** and **B**, Gene Ontology (GO) term enrichment visualized through bar/bubble plots (clusterProfiler). **C** and **D**, Kyoto Encyclopedia of Genes and Genomes (KEGG) pathway enrichment in bar/bubble charts (clusterProfiler).

Using the clusterProfiler package, we performed pathway enrichment analysis for *HAGH*, *OSBPL1A*, and *PANK2*. GO analysis revealed enrichment in biological processes, including bile acid metabolic processes, cellular modified amino acid metabolic processes, and cellular ketone metabolic processes (Figure 3A and B). KEGG analysis revealed enrichment in metabolic pathways, including pantothenate and CoA biosynthesis, pyruvate metabolism, and cofactor biosynthesis (Figure 3C and D).

### Exploring targeted signaling mechanisms associated with the key genes

**Figure 4:**
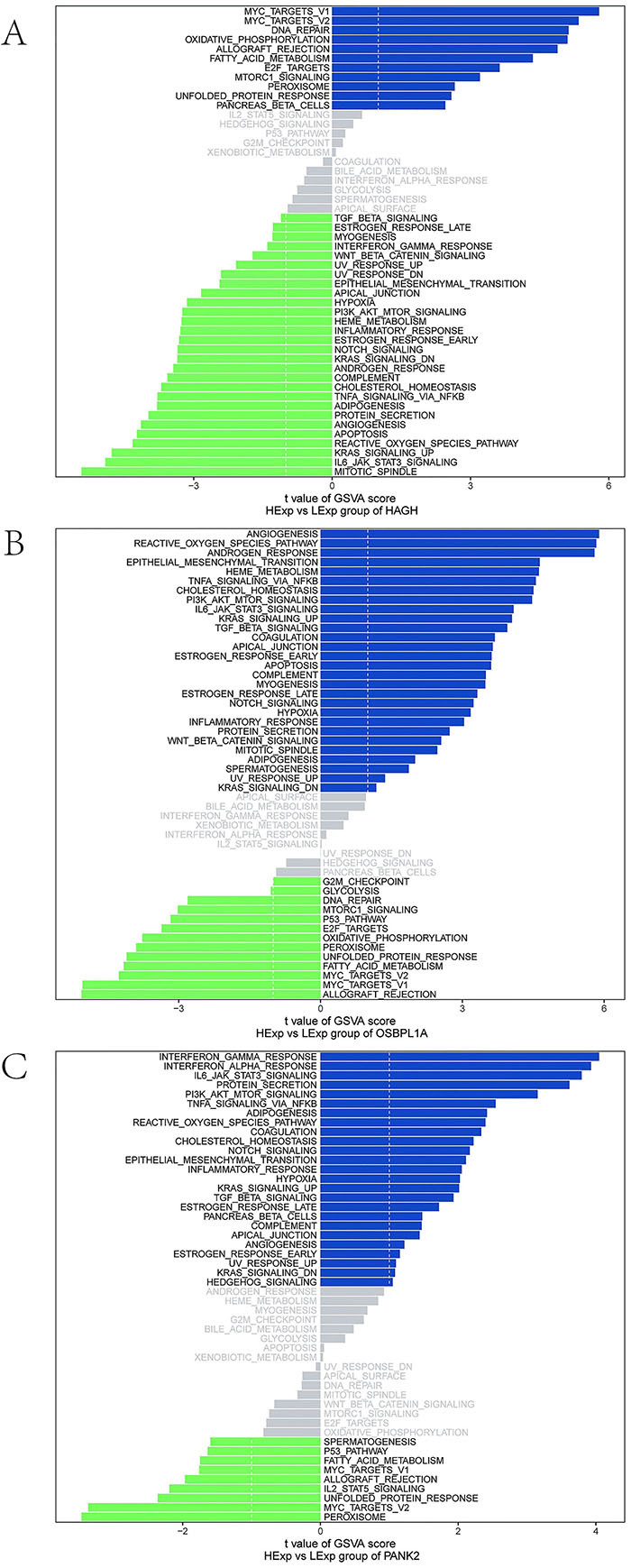
Gene set variation analysis (GSVA) of key genes. **A**-**C**, Pathway activation profiles: blue (high-expression associated), green (low-expression associated). Background gene set: Hallmark collection.

**Figure 5:**
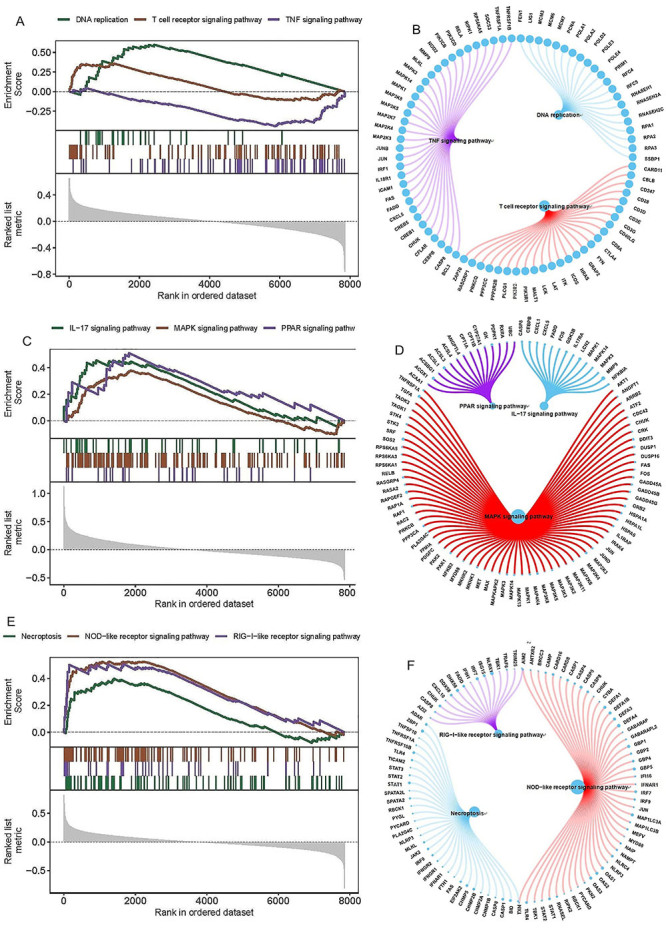
Gene set enrichment analysis (GSEA) of key genes. **A**-**F**, Enriched Kyoto Encyclopedia of Genes and Genomes (KEGG) pathways with coregulated gene networks.

To elucidate the molecular mechanisms by which *HAGH*, *OSBPL1A*, and *PANK2* influence epilepsy progression, we investigated their associated signaling pathways. GSVA revealed that *HAGH* was significantly enriched in the mTORC1 signaling and fatty acid metabolism pathways (Figure 4A). *OSBPL1A* was predominantly associated with apoptosis and ROS production pathways (Figure 4B). *PANK2* was associated with several pathways, including the PI3K/AKT/mTOR signaling pathway and the Notch signaling pathway (Figure 4C). GSEA revealed *HAGH* enrichment in DNA replication, T-cell receptor signaling, TNF signaling, and other pathways (Figure 5A and B). Similarly, *OSBPL1A* was enriched in the IL-17, MAPK, and PPAR signaling pathways (Figure 5C and D). *PANK2* was enriched in necroptosis, NOD receptor signaling, and retinoic acid-inducible gene I (RIG-I)-like receptor signaling pathways (Figure 5E and F). Collectively, these results suggest that *HAGH*, *OSBPL1A*, and *PANK2* may influence epilepsy progression by modulating these pathways.

### Expression levels of the key genes and disease-related genes

**Figure 6:**
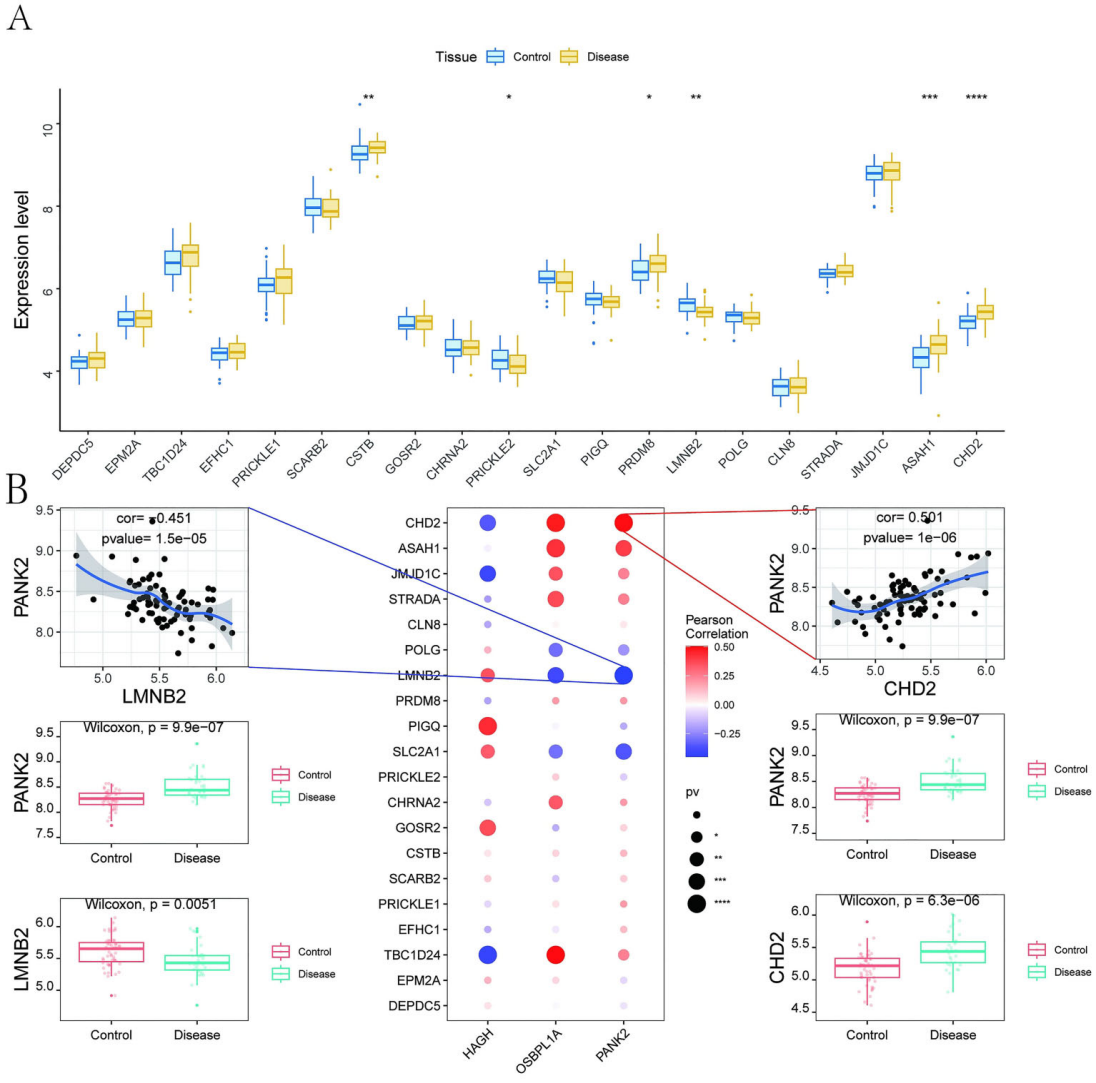
Key gene-disease regulator correlations. **A**, Differential expression of disease regulators: blue (controls), yellow (patients). **B**, Correlation heatmap: blue (negative), red (positive). Data are reported as median and interquartile range. *P<0.05; **P<0.01; ***P<0.001; ****P<0.0001 (Wilcoxon test).

Using the GeneCards database (https://www.genecards.org/), we identified epilepsy-associated genes and analyzed their expression profiles. Significant differences in the expression of *PRICKLE2*, *PRDM8*, *LMNB2*, *ASAH1*, and *CHD2* were observed between the groups (Figure 6A). Additionally, the expression levels of the three key genes were correlated with those of other genes. Specifically, *PANK2* was significantly positively correlated with CHD2 (correlation coefficient=0.501, P=1e-06) and significantly negatively correlated with *LMNB2* (correlation coefficient=-0.451, P=1.5e-05) (Figure 6B).

### Immune infiltration analysis

We further analyzed immune cell infiltration profiles (Supplementary Figure S4) and constructed a heatmap of immune cell correlation networks. In the heatmap, red indicates a positive correlation, blue indicates a negative correlation, and the color intensity reflects the correlation strength (Supplementary Figure S5A). Additionally, activated mast cell abundance was significantly greater in the disease group than in the control group (Supplementary Figure S5B). *PANK2* was significantly positively correlated with memory B cells and neutrophils and negatively correlated with gamma-delta T cells and naive B cells. *OSBPL1A* was positively correlated with neutrophils, M0 macrophages, and memory B cells and negatively correlated with naive B cells and activated CD4+ memory T cells. *HAGH* was positively correlated with activated CD4+ memory T cells and CD8+ T cells and negatively correlated with neutrophils and activated NK cells (Supplementary Figure S5C). We further queried the TISIDB database (20) for correlations between the key genes and immune factors, including immune modulators, chemokines, receptors, human leukocyte antigens, and immunosuppressants. The results revealed strong correlations between the key genes and immune factors (Supplementary Figure S6A-E). Collectively, these analyses demonstrated that the key genes are closely associated with immune cell infiltration levels and contribute to immune microenvironment regulation.

### Colocalization analysis

Colocalization analysis was performed for the three key genes at the eQTL-GWAS level. As shown in Supplementary Figure S7, the colocalization probabilities (PP.H4) for colocalized SNPs of the three key genes all exceeded 0.75, demonstrating a high degree of colocalization between gene expression regulation and disease-associated genetic signals. These results suggest that these genes may influence disease pathogenesis through their expression regulatory mechanisms, suggesting potential causal relationships. Colocalization analysis revealed that expression changes in these genes are likely causal drivers of disease rather than mere consequences, highlighting their potential causal roles in disease pathogenesis.

### Transcriptional regulation analysis

The key genes were selected as the gene set for this analysis, and their regulation by multiple transcription factors and shared regulatory mechanisms were investigated. Therefore, enrichment analysis of these transcription factors was performed via cumulative recovery curves. As shown in Supplementary Figure S8A-D, the motif with the highest normalized enrichment score (NES=5.88) was cisbp_M6518. All enriched motifs and their corresponding transcription factors for the key genes are presented.

### Nomogram analysis

The regression analysis results for the three key genes were visualized as a column chart according to their expression levels. Regression analysis revealed that varying expression distributions of the genes contributed differently to the overall scoring across all samples (Supplementary Figure S9A). Additionally, predictive analysis of the module performance demonstrated that the nomogram model exhibited good predictive accuracy (ROC=0.82) (Supplementary Figure S9B).

### ROC curve analysis

The predictive power of the key genes was evaluated via receiver operating characteristic (ROC) curves for diagnostic efficacy. Higher AUC values indicate better predictive performance. As shown in Supplementary Figure S9C-E, the AUC values for the three key genes were as follows: *PANK2* (0.815, 0.725-0.904), *HAGH* (0.657, 0.537-0.777), and *OSBPL1A* (0.692, 0.576-0.808). These results indicate that all three genes exhibit predictive utility for disease progression.

## Discussion

Epilepsy is widely recognized as a chronic disorder characterized by recurrent, transient, and stereotypical central nervous system dysfunction triggered by aberrant neuronal discharges (6). The hippocampus, a brain region highly susceptible to epilepsy, is referred to as the “pacing point” of epilepsy and remains the most intensively studied brain area in epilepsy research (6). Recurrent seizures induce pathological changes in brain tissue, particularly in the hippocampus, with neuronal apoptosis, necrosis, loss, glial cell proliferation, fiber sprouting, and hippocampal sclerosis being most pronounced (6). Accumulating evidence shows that epileptic seizures cause neuronal damage, with hippocampal neuronal injury being central to epilepsy pathogenesis. Thus, mitigating neuronal damage following epileptic seizures is critical for preventing seizure recurrence and progression (7). Mitochondria are essential organelles critical for maintaining neuronal physiological functions (9). Emerging evidence indicates that oxidative stress is central to epilepsy initiation and progression, with mitochondrial dysfunction emerging as a potential key mechanism in refractory epilepsy (21,22). The high mitochondrial content, high oxygen demand, and limited repair capacity of the brain render it particularly susceptible to oxidative damage. During epileptic seizures, abnormal neuronal discharges cause acute brain injury, inducing ROS accumulation and mitochondrial respiratory chain dysfunction (23). Mitochondrial dysfunction and impaired ROS clearance in the brain induce oxidative stress, which exacerbates epileptogenesis (23). During epileptic seizures in rats, neurons discharge abnormally. This causes deregulation of Ca^2+-^ and Ca^2+-^ binding proteins and a large amount of Ca^2+^ influx, resulting in aberrant activation of 1-methyl-4-phenyl-1,2,3,6-tetrahydropyridine (MPTP). This results in mitochondrial charge distribution dysregulation, reduced transmembrane potential, osmotic swelling, outer membrane rupture, structural damage, and functional impairment. Following cellular injury, mitochondrial matrix macromolecules (e.g., Cyto-C, Caspase-9, Apoptosis-inducing factor (AIF)) are released into the cytoplasm via the MPTP, initiating cell death pathways (23). Additionally, intracellular calcium overload places mitochondria in a high Ca^2+^ milieu, promoting oxygen free radical generation and neuronal damage via oxidative stress (23). This bidirectional MR study evaluated the causal relationship between mitochondrial dysfunction and epilepsy, with mitochondrial dysfunction promoting oxidative stress-mediated neuronal damage. The analysis revealed a clear causal association between genetic susceptibility to mitochondrial dysfunction and epilepsy onset. However, since the data were derived from a database limited to individuals of European descent, these findings should be interpreted as preliminary evidence highlighting the need for further studies with more diverse datasets to validate and enhance the generalizability of the results.

Three pairs of genes corresponding to positive eQTL outcomes were screened for causal relationships using the MR analysis. The identified genes were *HAGH*, *OSBPL1A*, and *PANK2*, respectively. Glyoxalase 2 (Glo2), encoded by the *HAGH* gene, belongs to the metallo-β-lactamase family and localizes to both the mitochondrial and cytoplasmic compartments (24). As the second enzyme in the glyoxalase system, it detoxifies α-ketothaldehyde methylglyoxal in cells. In addition to glyoxalase 1 (Glo1), glyoxalase 2 (Glo2) mediates the glyoxalase pathway in eukaryotic cells via the use of glutathione as a cofactor. Its biological importance is highlighted by its conserved presence in prokaryotic and eukaryotic organisms. While its canonical function in this system is well characterized, recent studies have revealed additional roles, particularly in oxidative stress responses (24). *OSBPL1A* belongs to the sterol sensor family and is implicated in lipid metabolism (25). Lipoic acid, an indispensable cofactor for mitochondrial metabolism, is generated through *de novo* pathways utilizing intermediates from mitochondrial fatty acid synthesis type II, S-adenosylmethionine, and iron-sulfur clusters. Epigenetic modifications are thought to play a key role in nutrient sensing. The human PANK2 protein, which functions as a homodimer, catalyzes the rate-limiting first step of CoA biosynthesis in a feedback-regulated manner (26). *PANK2* suppression results in reduced cell growth, upregulation of cell-specific ferroproteins, and iron dysregulation in human cell lines, phenocopying pathological features of neurodegenerative disorders with iron accumulation in the basal ganglia in the brain (26). Loss-of-function (LoF) mutations in *PANK2* impair mitochondrial function and disrupt iron homeostasis in mouse and human cells (26). Thus, *HAGH*, *OSBPL1A*, and *PANK2* regulate mitochondrial function through direct and indirect mechanisms. This study also revealed that *HAGH* was associated with reduced epilepsy risk, whereas *OSBPL1A* and *PANK2* were linked to increased epilepsy risk. Leave-one-out sensitivity analysis confirmed the robustness of the identified causal associations. However, the underlying mechanisms remain uncharacterized and warrant further investigation.

The clusterProfiler package was used to perform pathway enrichment analysis on *HAGH*, *OSBPL1A*, and *PANK2*. GO analysis revealed significant enrichment of genes involved in metabolic processes, including bile acid metabolism, modified amino acid metabolism, and ketogenic metabolism, suggesting their roles in modulating these metabolic pathways at the cellular and systemic levels. KEGG pathway analysis revealed significant enrichment of genes involved in the pantothenate and CoA biosynthesis, pyruvate metabolism, and cofactor biosynthesis pathways, all of which are critical for mitochondrial metabolism. Collectively, these findings indicate that *HAGH*, *OSBPL1A*, and *PANK2* play pivotal roles in regulating mitochondrial metabolic pathways.

We next delineated the signaling pathways associated with *HAGH*, *OSBPL1A*, and *PANK2* and their potential mechanistic roles in epilepsy pathogenesis. GSVA revealed a predominant association of *HAGH* with the mTORC1 signaling and fatty acid metabolism pathways. Both pathways critically regulate mitochondrial metabolism (27), and their dysregulation in astrocytes contributes significantly to epileptogenesis (27). *OSBPL1A* was significantly enriched in the apoptosis and ROS pathways, suggesting its modulatory role in epilepsy progression through these mechanisms. *PANK2* is linked primarily to the PI3K/AKT/mTOR and Notch signaling pathways. IL-1β-induced inflammation promotes seizure activity and contributes to temporal lobe epilepsy via the PI3K/AKT/mTOR and Notch signaling pathways (28,29). GSEA further revealed *HAGH* enrichment in DNA replication, T-cell receptor (TCR) signaling, and TNF signaling pathways. Errors in DNA replication/repair during neurodevelopment may cause somatic mosaicism, disrupting mTOR signaling and protein glycosylation, thereby contributing to cortical malformations and focal epilepsy (30). Increased CD3+/CD8+ T-cell infiltration in resected TLE hippocampi versus controls has been documented, although with intersubgroup heterogeneity (31). TNF-α is a primary upstream regulator of proinflammatory processes in LDs. Astrocyte-mediated TNF signaling activation induces necroptosis, neuroinflammation, and programmed necrosis (32). *OSBPL1A* enrichment was observed in the IL-17, MAPK, and PPAR signaling pathways. The IL-17 and TNF signaling pathways play pivotal roles in blood-brain barrier disruption and central nervous system autoinflammation, which are closely linked to seizures (33). The P38 MAPK pathway mediates cognitive impairment in pentylenetetrazole-induced epilepsy through the apoptotic cascade (34). Peroxisome proliferator-activated receptor gamma (PPARγ) activation mitigates ferroptosis-induced hippocampal damage via Nrf2/GPX4 signaling in epileptic rats (35). *PANK2* was enriched in necroptosis, NOD-like receptor, and RIG-I-like receptor signaling pathways. Necroptosis is a type of controlled cell death that has been associated with epilepsy (36). A decrease in the activity of the NLRP3 inflammasome alleviates epileptic seizures and neuroinflammation (37). RIG-I-like receptors drive the polarization of inflammatory macrophages in response to West Nile virus infection (38). Notably, RIG-I-like receptor involvement in epilepsy remains unreported. Collectively, these genes modulate epilepsy via inflammation, immunity-, metabolism-, apoptosis-, and necroptosis-related pathways. The precise mechanism requires further validation.

Using the GeneCards database (https://www.genecards.org/), we identified epilepsy progression-associated genes. Differential expression analysis of the top 20 relief-scored genes revealed significant alterations in the following genes between the patient and control groups: cystatin B (*CSTB*), prickle-like protein 2 (*PRICKLE2*), PR domain zinc finger protein 8 (*PRDM8*), lamin B2 (*LMNB2*), N-acylsphingosine amidohydrolase 1 (*ASAH1*), and chromodomain-helicase-DNA-binding protein 2 (*CHD2*). Significant correlations were detected between these key genes and established progression-associated markers. *PANK2* expression was positively correlated with *CHD2* but inversely correlated with *LMNB2*. *CHD2* pathogenic variants are documented in early-onset epilepsy cohorts. Among reported cases, 63% (25/40) develop developmental and epileptic encephalopathies (DEEs). DEE encompasses early-onset epilepsies characterized by refractory seizures and seizure-associated cognitive decline/regression. *LMNB2* localizes to mitochondria, maintaining energy production via mitochondrial function (38). *LMNB2* is expressed at high levels during oligodendrocyte maturation and supports glial cell development/maintenance (39). *LMNB2* critically regulates neuronal migration, survival, and function in the nervous system. The *LaminB2* p. His157Tyr variant is associated with progressive myoclonic epilepsy (PME) (39).

The tumor microenvironment (TME) comprises fibroblasts, immune cells, the extracellular matrix, growth/inflammatory factors, and physicochemical features (40). The TME influences disease diagnosis, survival outcomes, and therapeutic responses. These key genes are correlated with immune cell infiltration and cytokine profiles, indicating their functional significance in the immunomodulatory microenvironment. Modulating *HAGH*/*OSBPL1A*/*PANK2* expression may remodel the immunogenic niche and improve epileptic prognosis.

Colocalization analysis revealed that *HAGH*/*OSBPL1A*/*PANK2* are involved in epileptogenesis, suggesting causal roles in disease pathogenesis. Nomogram-ROC analysis demonstrated robust predictive efficacy for epilepsy prognosis. High AUC values confirmed strong predictive capacity for epileptogenesis and progression.

This study has two principal limitations. First, the reliance on public databases limits the inclusion of clinical parameters detailing the relationships between mitochondrial dysfunction and epilepsy. Second, the exclusive use of European retrospective cohorts constrains population generalizability. Future prospective clinical studies with mechanistic validation (*in vitro*/*in vivo*) are warranted.

In summary, we identified *HAGH*, *OSBPL1A*, and *PANK2* as key regulators of epilepsy pathogenesis. Mitochondrial dysfunction represents a recognized epileptogenic mechanism. These genes potentially modulate epilepsy through mitochondrial signaling pathways and show strong predictive capacity for epileptogenesis. These findings reveal candidate molecular targets for clinical intervention and biomarkers for tracking epileptogenic progression. Current findings preliminarily characterize mitochondrial dysfunction-mediated epileptogenic mechanisms. Experimental validation using *in vitro* neuronal models and *in vivo* epileptogenesis models is essential.

## Data Availability

All data generated or analyzed during this study are included in this published article.

## Supplementary Materials

Supplementary Material Click to view [pdf].
