# Supplementary material for: Exploring the causal impact of mitochondrial dysfunction on epilepsy: a mendelian randomization study
Source: Braz J Med Biol Res. 2026 Mar 9;59:e14524. doi: 10.1590/1414-431X2025e14524 (PMC12971012; doi:10.1590/1414-431X2025e14524)

Figure S1. Overview of the study design. Schematic representation of core epilepsy-associated gene identification and mechanistic exploration via weighted gene coexpression network analysis (WGCNA), mitochondrial gene screening, Mendelian randomization, functional enrichment analysis (clusterProfiler), gene set enrichment analysis (GSEA), gene set variation analysis (GSVA), and correlation analysis.

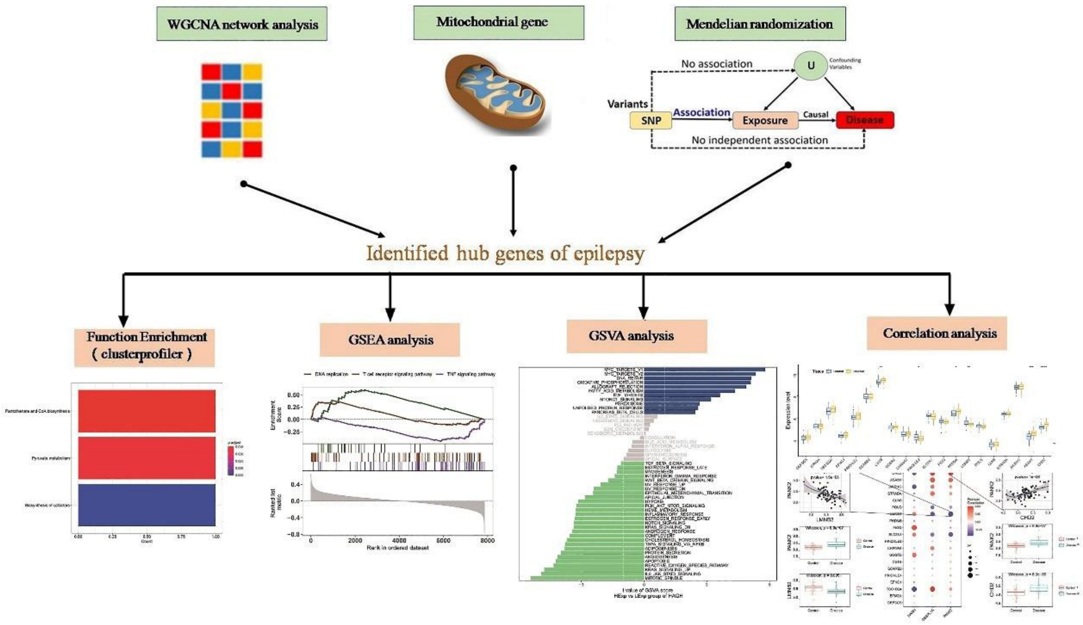

Figure S2. Leave-one-out analysis. **A–C**, Funnel plots assessing bias for key genes. **D–F**, Forest plots of single-nucleotide polymorphisms (SNP)-specific effects.

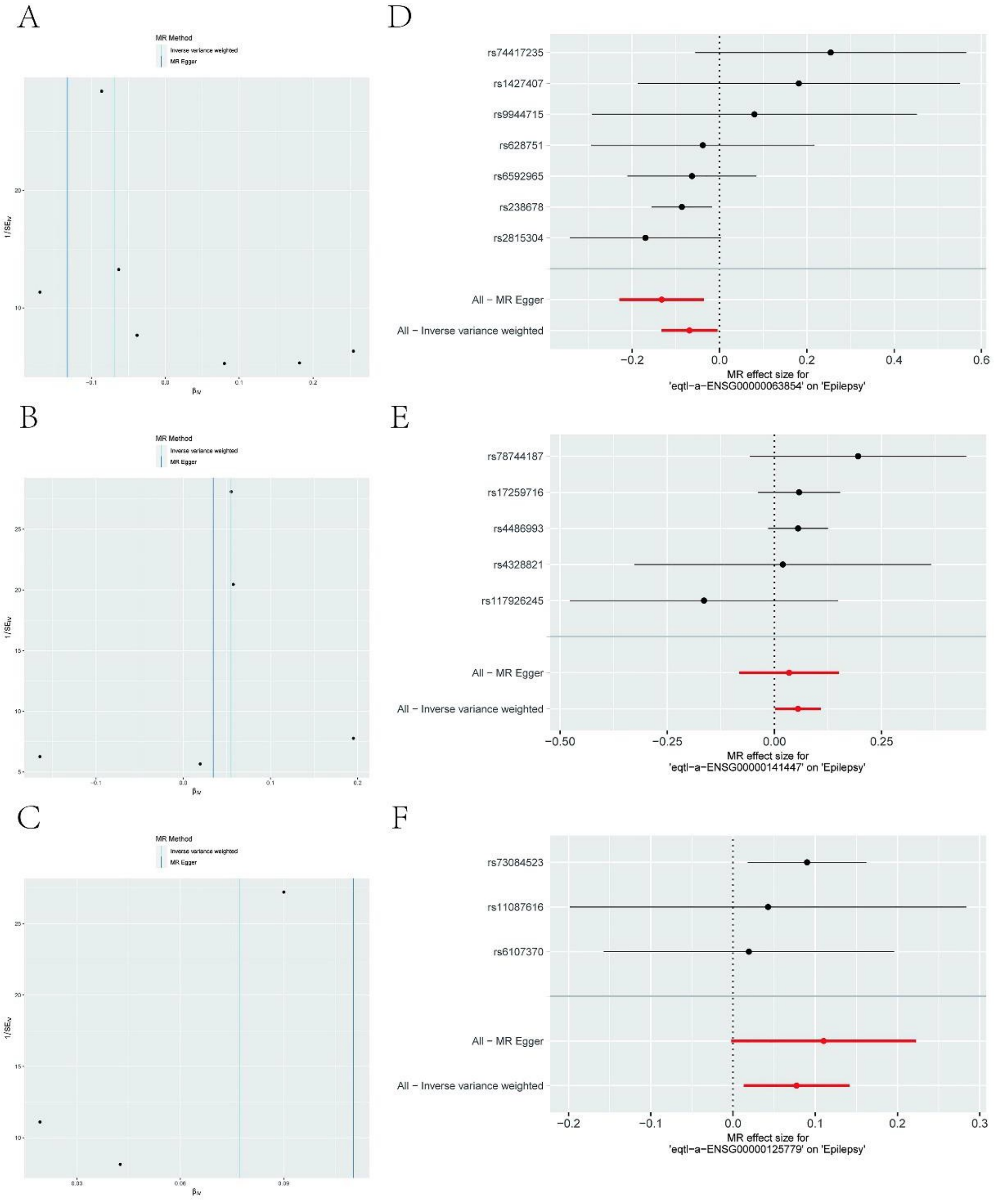

Figure S3. Heterogeneity assessment via leave-one-out analysis. **A**–**C**, Sequential exclusion of individual genetic variants to evaluate causal effect robustness on outcome variables.

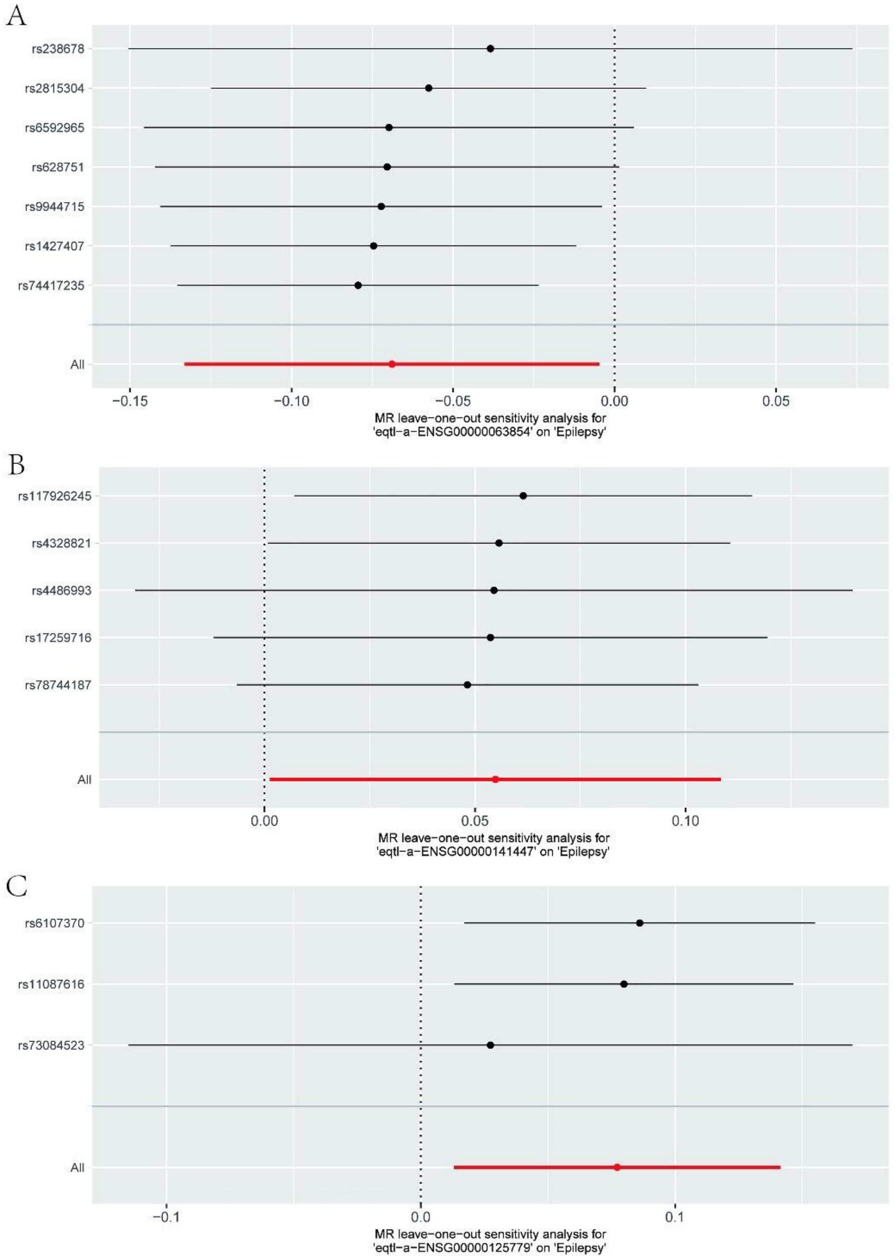

Figure S4. Immune cell composition. Relative abundances of 22 immune cell subtypes.

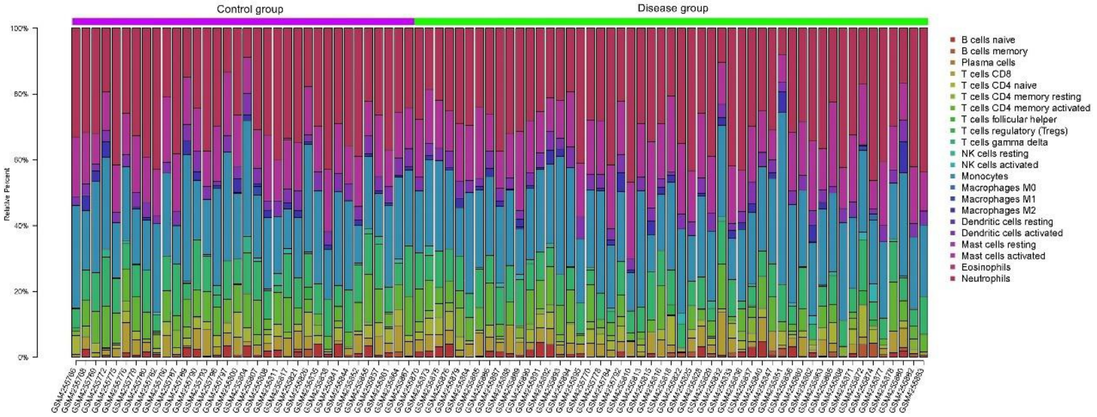

Figure S5. Key gene-immune infiltration correlations. **A**, Immune cell intercorrelation matrix: blue (negative), red (positive). **B**, Differential immune cell abundances: blue (controls), yellow (patients). **C**, Key gene-immune cell correlations: blue (negative), red (positive). Data are reported as median and interquartile range. *P<0.05; **P<0.01; ***P<0.001 (Wilcoxon test).

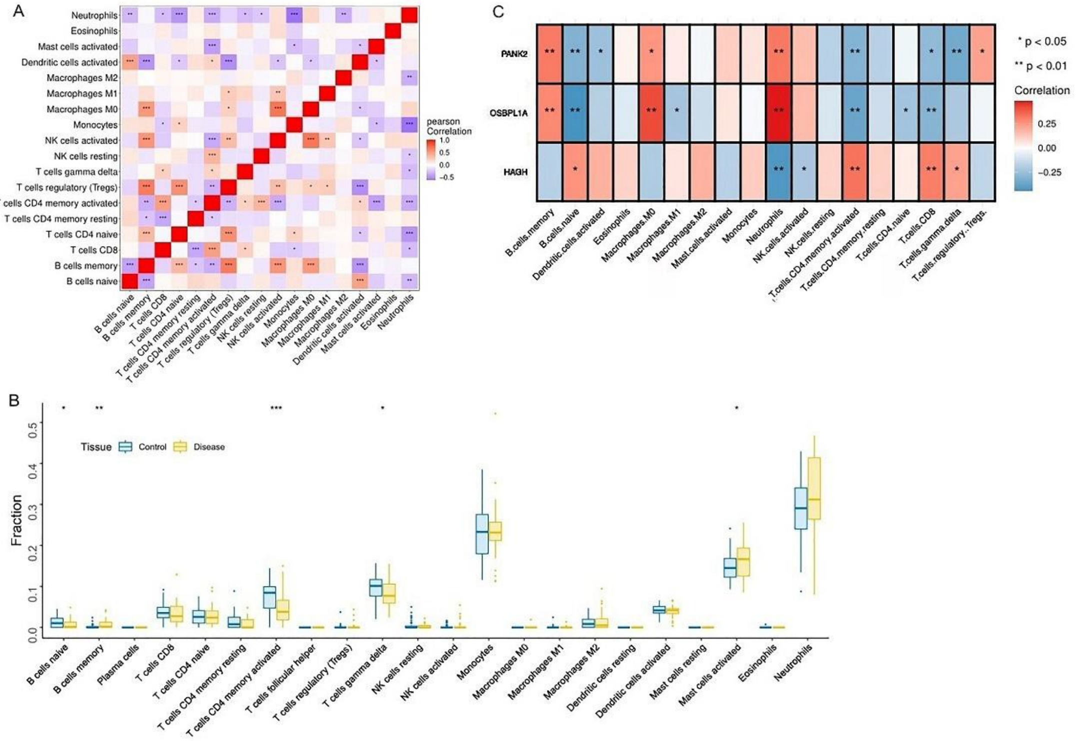

Figure S6. Key gene-immunomodulator correlations. **A**, Chemokines; **B**, immunosuppressors; **C**, immunostimulators; **D**, MHC molecules; and **E**, receptors. Color scheme: blue (negative), red (positive).

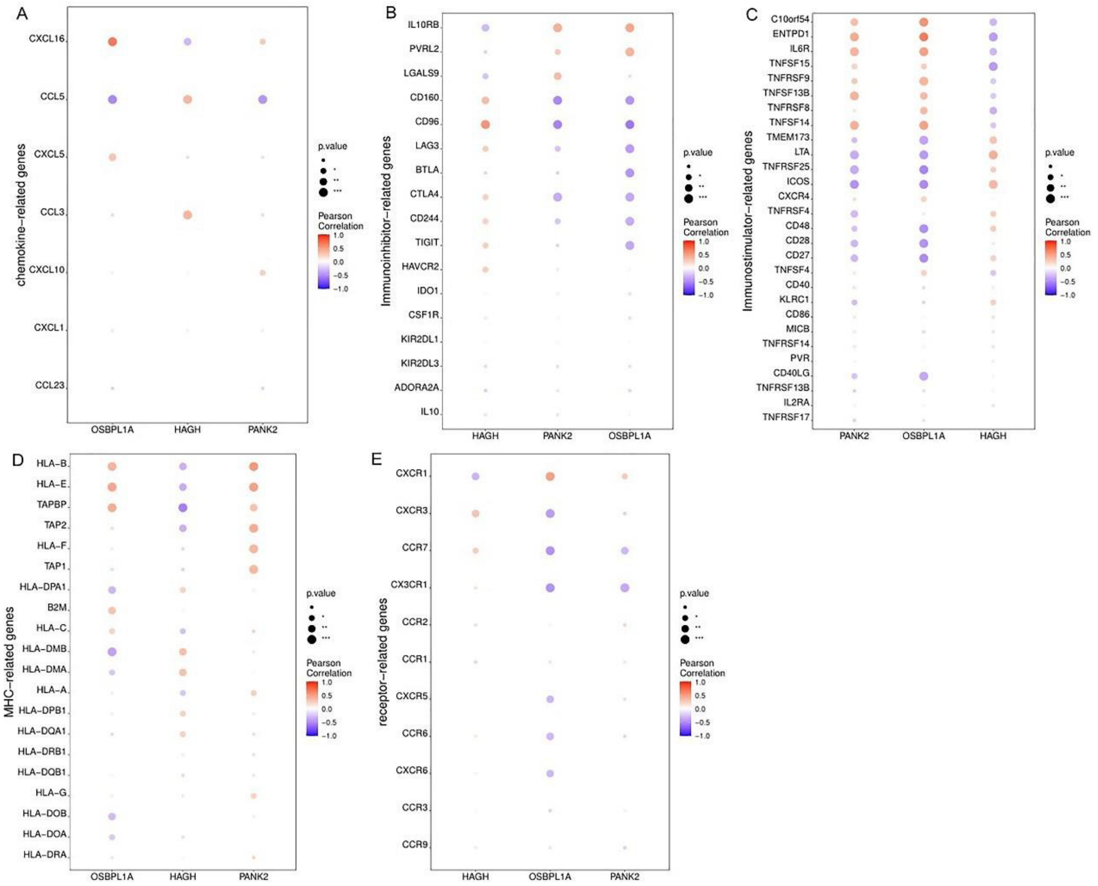

Figure S7. eQTL-GWAS colocalization of key genes. **A**, *HAGH*; **B**, *OSBPL1A*; and **C**, *PANK2*.

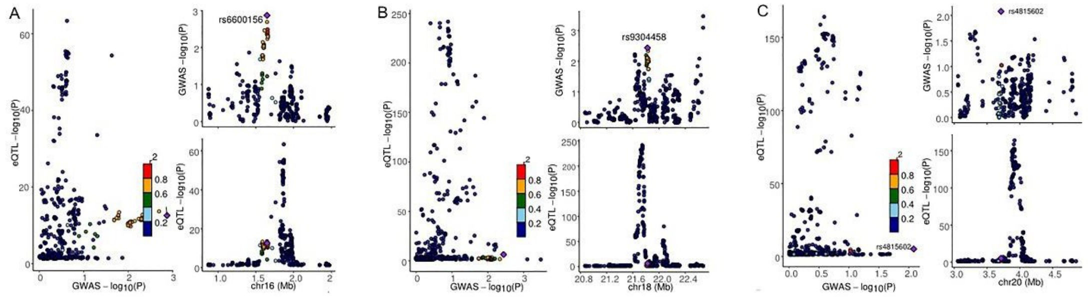

Figure S8. Transcriptional regulation analysis. The top area under the curve (AUC) motifs are shown in red (mean recovery curve), green (means±SD), and blue (current motif curve). Maximum enrichment level determined at peak separation between the current motif and the green curve (means+SD). Insets: individual motifs. The top three motifs according to the normalized enrichment score (NES) are displayed.

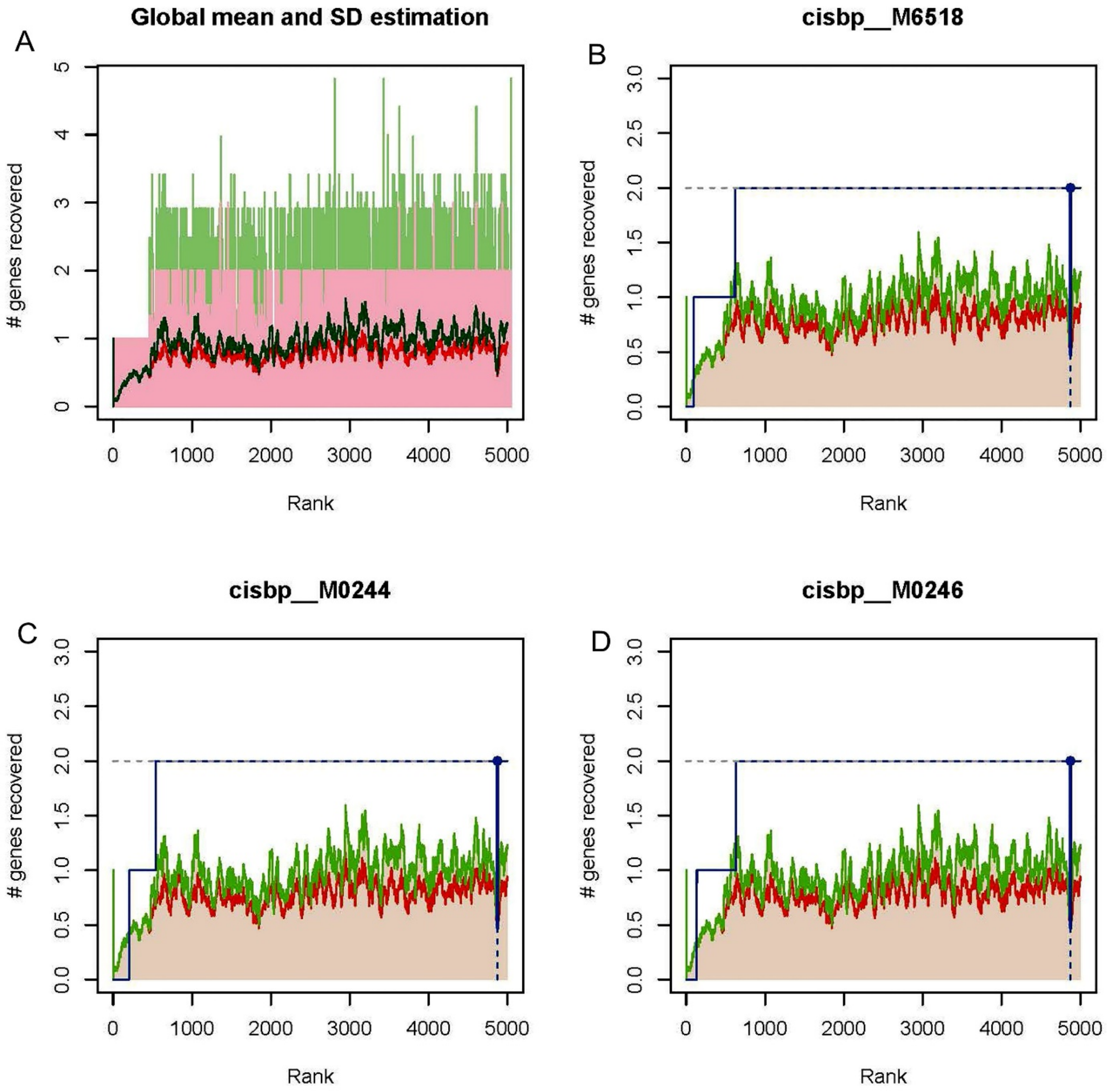

Figure S9. Nomogram and receiver operating characteristics (ROC) analysis. **A**, Key gene expression regression coefficients. **B**, Nomogram performance validation. **C**, *PANK2* ROC curve (area under the curve, AUC). **D**, *HAGH* ROC curve (AUC). **E**, *OSBPL1A* ROC curve (AUC).

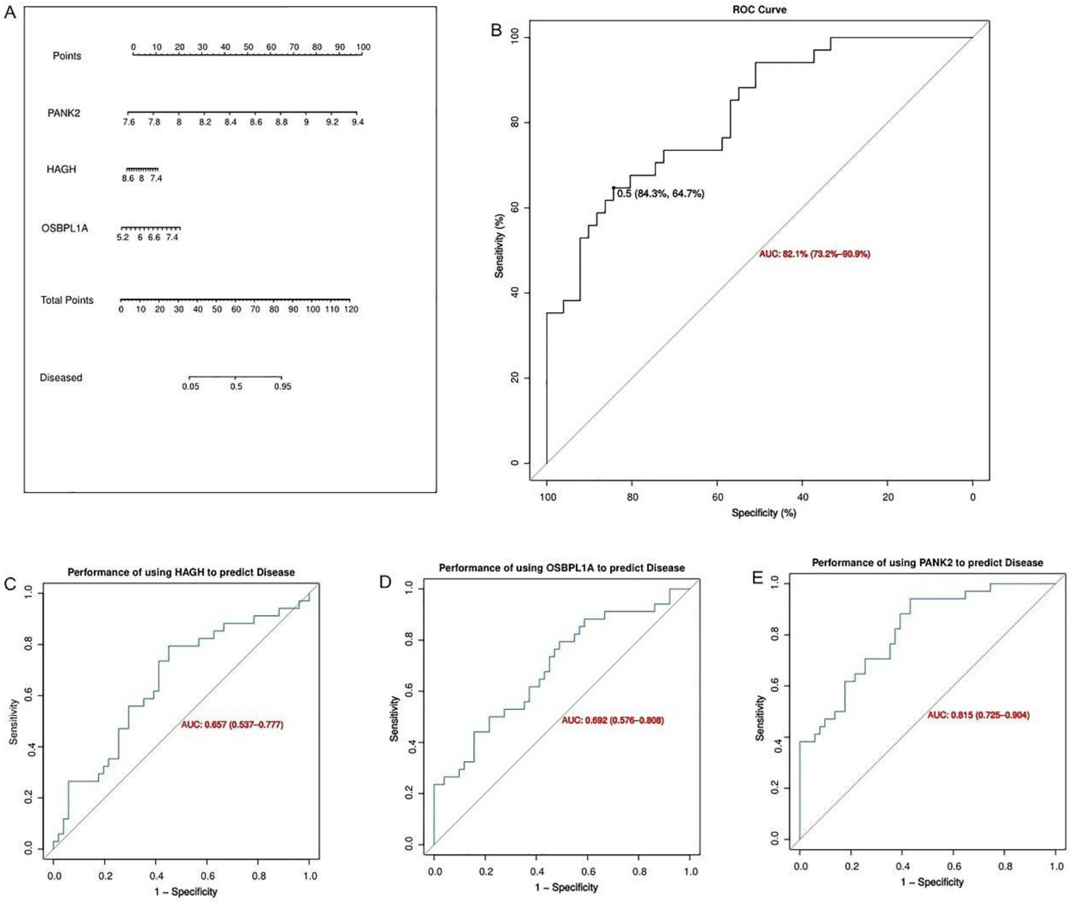

Supplement: Supplementary Material [file 1414-431X-bjmbr-59-e14524-suppl.pdf]
